# Binding of Features and Responses in Inhibition of Return: The Effects of Task Demand

**DOI:** 10.5334/joc.247

**Published:** 2022-11-10

**Authors:** Hsuan-Fu Chao, Fei-Shan Hsiao, Shih-Ching Huang

**Affiliations:** 1 Department of Psychology, Chung Yuan Christian University, Taoyuan City, Taiwan

**Keywords:** binding, event file, inhibition of return, task demand

## Abstract

Binding of target’s location and response has been demonstrated in inhibition of return (IOR). This study further investigated the effects of task demand on the binding of the target’s form to the target’s location and response in the target-target paradigm of IOR. Experiments 1 (detection task) and 2 (localization task), in which the target’s form was task irrelevant, suggested the binding of location and response. Experiment 3 (discrimination task), in which the target’s form was task relevant, showed the binding of target’s form, location, and response. These findings support the concept that the features and responses associated with a target are integrated into episodic representations or event files for the target event. Furthermore, task demand modulates the binding or retrieval of the event files.

Inhibition of return (IOR) is the phenomenon in which it takes longer for people to respond to a target presented at a previously cued location than to one at an uncued location (Posner & Cohen 1984; Posner et al. 1985; see Klein 2000, for a review). Several hypotheses have been proposed to explain this phenomenon. According to the attentional inhibition hypothesis, IOR reflects the aftereffect of attentional inhibition of previously attended spatial locations (Posner & Cohen 1984; Posner et al. 1985). As the attended location is initially activated and then inhibited, time is required to overcome the lingering inhibitory effect to respond to a target at this cued and attended location. According to the constructive retrieval hypothesis, when a target is presented at the cued location, this perceptual event is integrated with the episodic representation of the previous cue event due to their spatiotemporal proximity. A new episodic representation is established when a target is presented at an uncued location. Therefore, the cuing effect depends on the relative efficiency of these two processes: integration with old representations and establishment of new representations (Milliken et al. 2000). Moreover, according to the habituation hypothesis, IOR reflects the habituation of spatial orientation (Dukewich 2009). Finally, according to the detection cost hypothesis, IOR demonstrates the difficulty of detecting a target that is similar to the episodic representations of the previous cue (Lupiáñez 2010; Lupiáñez et al. 2007; Lupiáñez, Martín-Arévalo & Chica 2013; Martín-Arévalo, Kingstone & Lupiáñez 2013). The constructive retrieval, habituation, and detection cost hypotheses suggest that memory representations of cue events, rather than attentional inhibition, play an essential role in IOR.

Episodic representations, or object files (Kahneman, Treisman & Gibbs 1992), of cue events are essential in both the constructive retrieval and detection cost hypotheses of IOR (Lupiáñez, Martín-Arévalo & Chica 2013; Milliken et al. 2000). The cuing effect is related to the integration of the target into the object files of the cue event. Additionally, when the repetition of responses is taken into account, such as in the target-target paradigm of IOR (Maylor & Hockey 1985), the operation of event files (Hommel 1998, 2004) should be further considered (Lupiáñez, Martín-Arévalo & Chica 2013). In the target-target paradigm of IOR (Maylor & Hockey 1985), participants respond to the first target, followed by a second target. These two targets can be presented at the same or different locations. Slower responses in the same-location condition than in the different-location condition revealed an IOR effect. In this paradigm, responses to the target may also be integrated into the episodic representations of the first target, hence influencing the processing of the second target. Thus, the idea of event files (Hommel 1998, 2004), which further proposes that actions and stimulus features are bound together, can account for the underlying processes in the target-target paradigm of IOR.

Following the idea of event files (Hommel 1998, 2004), it is assumed that the responses to the target event are integrated into episodic representations of the first target in the target-target paradigm of IOR. When some or all of the features associated with the first target (e.g., location, form, and response) were repeated in the second trial, the episodic representation of the first trial was retrieved. Retrieved event files or episodic representations would affect the processing of the second target. Therefore, it was predicted that response repetition would affect the processing of the second target. According to the framework of transfer-inappropriate and transfer-appropriate processing (Neill 2007; Neill & Mathis 1998), a repetition benefit is expected when the retrieved response is compatible with the current response. Furthermore, a repetition cost is predicted when the retrieved response is incompatible with the current response. To examine these predictions, Chao and Hsiao (2021) combined Hommel’s (1998) response-cuing procedure with a target-detection task, demonstrating the effect of response repetition in the target-target paradigm of IOR. When the response to the first target was not repeated as a response to the second target, the IOR surfaced in the location repetition condition. When the responses were repeated, facilitation of return (FOR: benefit of location repetition) was observed. The interaction between location repetition and response repetition suggests that, in the target-target paradigm of the IOR, the responses to the target are integrated into the object file of the first target. Therefore, the effect of location repetition on performance is contingent on whether the response is repeated. These findings are consistent with predictions based on the concept of event files (Hommel 1998, 2004) and the framework of transfer-inappropriate and transfer-appropriate processing (Neill 2007; Neill & Mathis 1998).

It should be noted that although Chao and Hsiao (2021) demonstrated the integration between responses and spatial locations in the target-target paradigm of IOR, they did not observe an integration between the visual forms of the targets and responses and spatial locations. For instance, the effects of location and response repetition were not modulated by form repetition, implying that the visual form may not be integrated with the location of and response to the target. A potential explanation for this is the effect of task demand. In that study, the visual forms of the targets were task-irrelevant features. Thus, they may be less likely to be integrated into the event files and less likely to be retrieved from the event files. According to Memelink and Hommel (2013), an intentional weighting mechanism influences perception and action. The weighting mechanism assigns task-relevant dimensions (e.g., color, orientation, or location) more weight than task-irrelevant dimension (see also Hommel et al., 2001). Hence, task demand affects the processing of event files during the retrieval stage (Hommel 2019; Hommel et al. 2014). Moreover, according to Frings et al.’s (2020) framework of binding and retrieval in action control (BRAC), top-down control, such as attentional weighting, affects encoding (binding) as well as retrieval. Hence, the task demand might affect what was encoded into and retrieved from the event files. Consistent with these accounts, empirical evidence supports the effects of task demand or task relevance on binding to or retrieval from event files (Mocke et al. 2020; Moeller & Frings 2014; Singh et al. 2018). In their study on action planning, Mocke et al. (2020) showed that task-relevant features were bound to action plans, whereas task-irrelevant features were not. Moeller and Frings (2014) used a prime-probe paradigm and showed that attended stimuli modulated the retrieval of event files. Additionally, Singh et al. (2018) instructed participants to either attend to the valence or word type of the stimuli. Their findings also suggested that attention modulates binding or retrieval. Consistent with these theoretical accounts and empirical findings, Huffman, Hilchey and Pratt’s (2018) review of the attentional-orienting literature indicated that there was no clear evidence for non-spatial feature integration in detection and localization tasks when the non-spatial features were task-irrelevant. Therefore, it was reasonable to expect that in the target-target paradigm of IOR, a non-spatial feature was more likely to be integrated into (or retrieved from) the event files when discrimination of this non-spatial feature was required. Consistent with this, Schöpper et al. (2020) showed that color and location were bound together when the task was color discrimination and did not integrate when the task was target detection.

The present study investigated the hypothesized effects of task demand or task relevance on the binding of non-spatial features to the locations and responses in the target-target paradigm of IOR. This was conducted following the findings that responses and spatial locations were bound in a detection task in IOR’s target-target paradigm. Furthermore, the forms were not integrated with the response and the location (Chao & Hsiao, 2021). Detection, localization, and discrimination tasks were used in Experiments 1, 2, and 3, respectively. We expected that in Experiment 3, in which form discrimination of the target was required, the form would more likely be integrated with the locations and responses and retrieved to modulate the effects of IOR.

## Experiment 1: Detection Task

In this study, the target-target paradigm of the IOR was used. The couplets consisted of two successive trials (the first and the second trial). Whether the location and form of the target of the first trial and the response to this first target were repeated in the second trial were orthogonally manipulated.

In Experiment 1, a task similar to that of Chao and Hsiao (2021) was used to manipulate whether the response was repeated, combining Hommel’s (1998) response-cuing procedure with the target detection task. It was expected that, similar to Chao and Hsiao (2021), evidence for the integration of location and response should be observed. For instance, the IOR should surface when the response is not repeated, and FOR should manifest when the response is repeated in a couplet.

Additionally, according to Frings et al.’s (2020) BRAC framework, retrieving event files is critical for the effects of the event files to manifest. Furthermore, according to Chao and Hsiao’s (2021) analysis of the vincentized cumulative response time (RT) distribution (Ratcliff 1979), the IOR surfaced when RTs were relatively long. This finding implies that it may take time for the event files of target events to be retrieved. Hence, we analyzed the vincentized cumulative RT distribution in the present study.

Finally, the form of the target (rectangle or oval) was repeated within a couplet and was manipulated in this experiment. Considering the intentional weighting mechanism (Memelink & Hommel 2013) and the effects of task demand on the processing of event files (Hommel 2019; Hommel et al. 2014), it was expected that repetition of the form would be less likely to affect performance in the present experiment because the form of the target was not related to the task demand (target detection).

### Methods

#### Participants

A total of 20 undergraduate students at Chung Yuan Christian University (6 men and 14 women, aged 20–25 years old, average age = 21.1 years) participated in this experiment and received NT$320. According to a Monte Carlo simulation (Lakens & Caldwell 2019), for the critical two-way interaction between location repetition and response repetition reported by Chao and Hsiao (2021), the power was 0.99 for a sample size of N = 20 and an alpha of 0.05. Therefore, the sample size was 20 in all experiments in the present study.

Each participant had a normal or corrected-to-normal vision. The Research Ethics Committee of the National Taiwan University (201512ES045) reviewed and approved this study. All participants provided written informed consent to participate in the study.

#### Stimuli

In this experiment, the viewing distance was approximately 57 cm. At the beginning of each trial, three boxes were presented at the center of the screen as placeholders, aligned on the horizontal meridian. The box’s outline was presented in white, with a thickness of 0.1°. Each box had a width of 3.0° and a height of 2.0°. The center-to-center distance between the left and right boxes and the central box was 4.8°. When the central cue was presented, the width of the central outline increased to 0.3°, and a Chinese word indicating left or right was presented at the center of this central box as the response cue. The height and width of the Chinese words were 0.8°. The target was a rectangle or oval, presented in the center of the left or right box. The length and width of the rectangle and oval were 1.1° and 0.7°, respectively. The target was always presented in yellow and vertically.

#### Procedure

This experiment was conducted using the DMDX software (Forster & Forster 2003). Given the use of the target-target paradigm of IOR, couplets comprising two successive trials (first and second trials) were used in the present experiment.

There was a practice block of 16 test couplets and four catch couplets at the beginning of the experiment, followed by 384 test couplets and 96 catch couplets. In the test couplets, a target was present in both the first and second trials. The location required response and form of the first target may be repeated in the second trial, resulting in a location repetition (repeated/unrepeated) × form repetition (repeated/unrepeated) × response repetition (repeated/unrepeated) design. There were 48 couplets for each combination of these three variables. In the catch couplets, the target was present in the first trial but absent in the second trial (32 couplets), absent in the first trial but present in the second trial (32 couplets), or absent in both the first and second trials (32 couplets).

**Figure 1:**
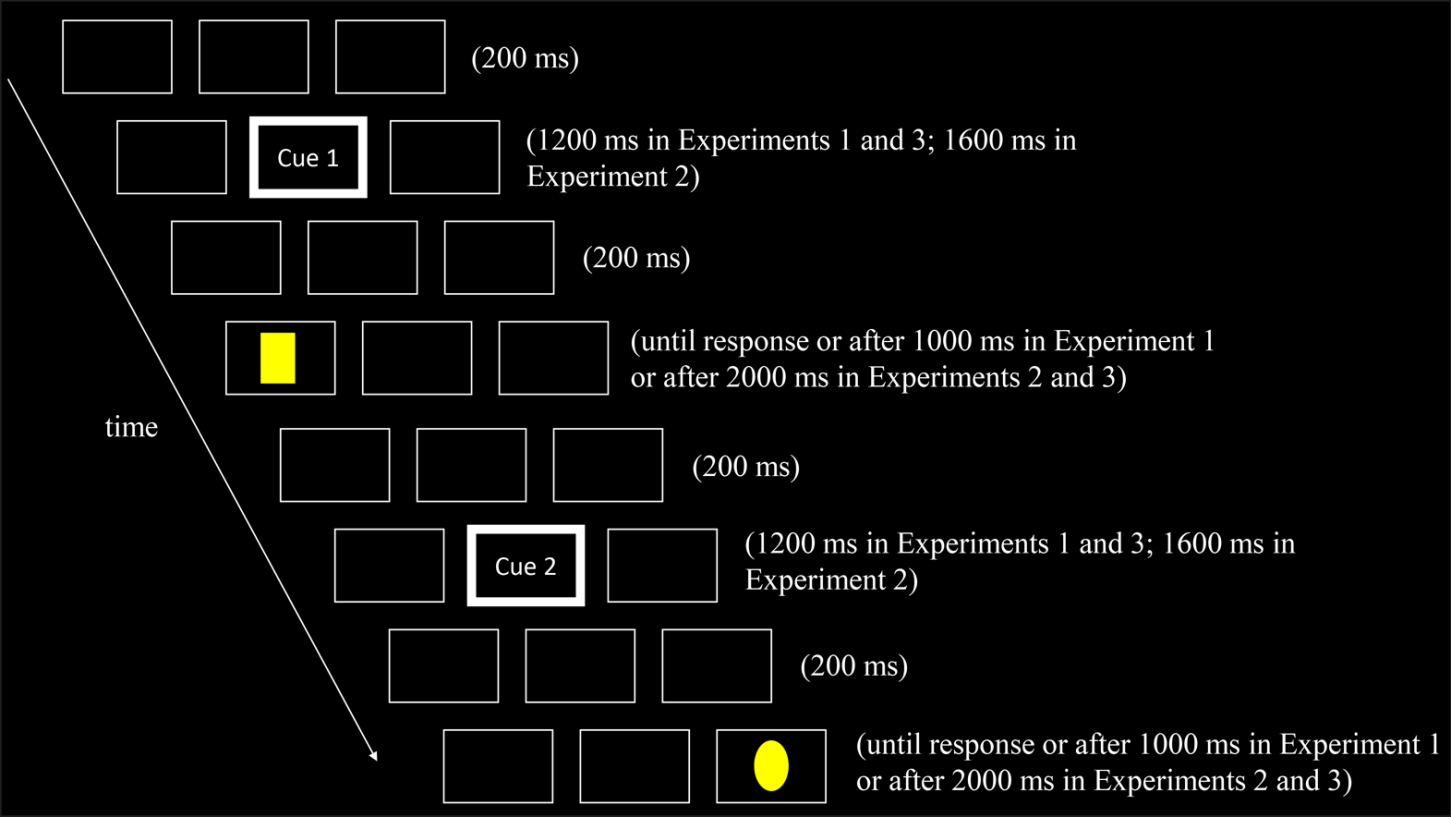
An Example of a Couplet (Not to Scale). *Note*: Two trials were performed for each couplet. Each trial began by displaying three boxes as placeholders for 200 ms. Subsequently, the width of the outline of the central box was increased as a fixation cue for 1200 ms (Experiments 1 and 3) or 1600 ms (Experiment 2). During the same period, a response cue indicating the response to make was presented in the central box. In Experiment 1, Cue 1/Cue 2 was a Chinese word indicating the left or right. In Experiment 2, Cue 1/Cue 2 were Chinese words indicating ‘press the left button for a target at the left location; press the right button for a target at the right location’ or ‘press the right button for a target at the left location; press the left button for a target at the right location’. In Experiment 3, Cue 1/Cue 2 were Chinese words indicating that ‘press the left button for an oval; press the right button for a rectangle’ or ‘press the right button for an oval; press the left button for a rectangle’. After an interval of 200 ms, a target was presented in either the left or right box, or the target was absent.

Figure 1 illustrates an example of a couplet. At the beginning of each trial of each couplet, the placeholders were presented for 200 ms, followed by a 1200-ms central cue. After another interval of 200 ms, only the placeholders were presented. Further, a target was presented in the left or right box for 1,000 ms or until a response was given. Participants were instructed to press the mouse button specified by the central cue at the beginning of each trial upon seeing a target, regardless of the target’s form and location. The target was absent in the first, second, or both trials in catch couplets. The participants rested after completing each block of 30 couplets.

### Results

Responses to the second trial of each couplet were included in further analyses only when the responses to the first trial of that couplet were correct. This criterion excluded 1.6% of the data (0%–4.2% per participant).

### Analysis of the overall data

Table 1 lists each condition’s average median correct RTs and error rates. Both RTs and error rates were analyzed using location repetition (repeated/unrepeated) × form repetition (repeated/unrepeated) × response repetition (repeated/unrepeated) repeated-measures analysis of variance (ANOVA).

**Table 1:** Averaged Median Correct Response Times (RT, in ms) and Error Rates (ER, %) and Standard Deviations (in parentheses) as a Function of Location, Form, and Response Repetition in Experiment 1 (Detection Task).


	LOCATION REPEATED				LOCATION UNREPEATED			
	
	FORM REPEATED		FORM UNREPEATED		FORM REPEATED		FORM UNREPEATED	
			
	RESPONSE REPEATED	RESPONSE UNREPEATED	RESPONSE REPEATED	RESPONSE UNREPEATED	RESPONSE REPEATED	RESPONSE UNREPEATED	RESPONSE REPEATED	RESPONSE UNREPEATED

RT	317(39)	338(47)	313(33)	331(47)	334(42)	324(42)	325(47)	317(33)

ER	0.7(1.4)	1.7(2.1)	0.9(1.9)	1.5(2.5)	1.1(1.8)	1.2(2.0)	2.8(3.0)	1.5(2.3)


**Figure 2:**
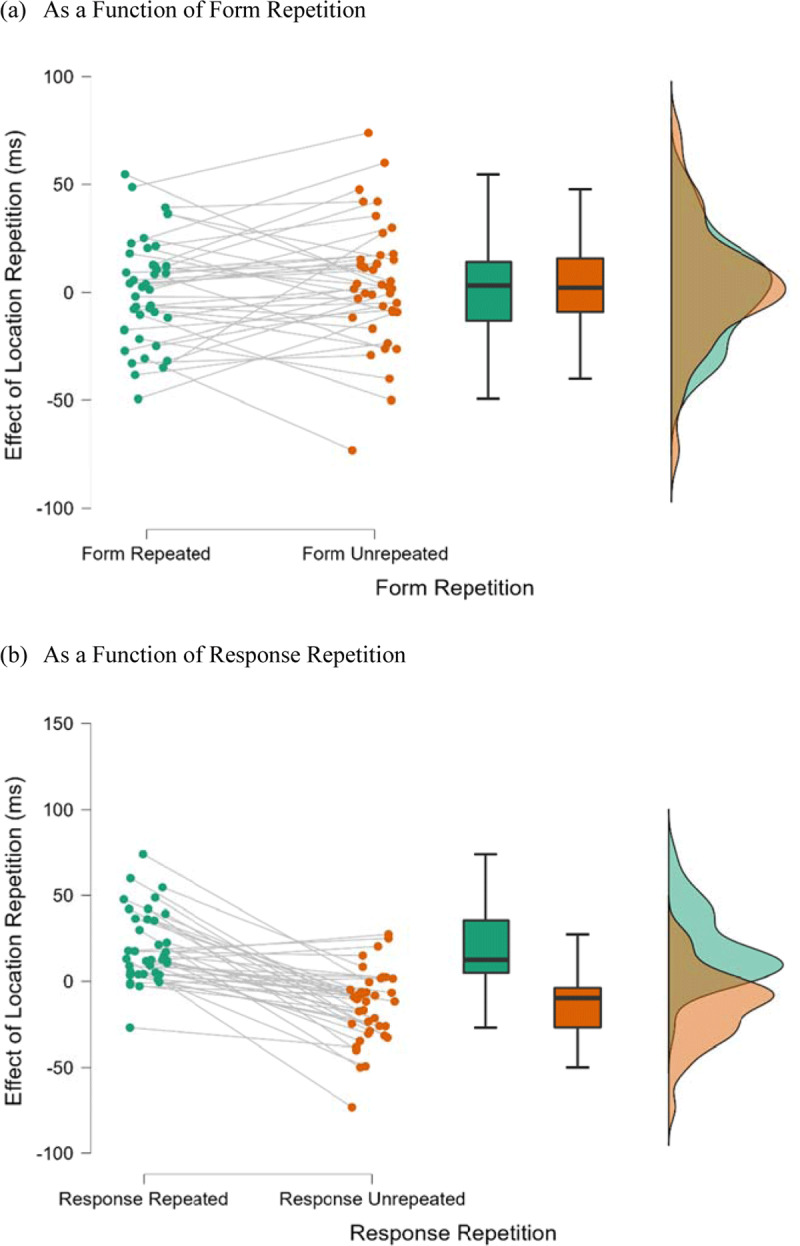
Raincloud Plots for the Effect of Location Repetition as a Function of Form Repetition and Response Repetition in Experiment 1 (Detection Task). *Note*: Effect of location repetition = unrepeated-location RT – repeated-location RT.

Analysis of the RT data revealed a significant interaction between location repetition and response repetition, *F*(1, 19) = 40.69, *p* < 0.001, \eta _{\rm{p}}^2 = 0.68. Figure 2 shows the raincloud plots (Allen et al., 2021) for the effect of location repetition as a function of response repetition and form repetition. A follow-up simple main effect analysis indicated that location repetition produced a significant repetition benefit when the response was repeated (19 ms), *F*(1, 38) = 26.97, *p* < 0.001, \eta _{\rm{p}}^2 = 0.42, and a significant repetition cost when the response was not repeated (–14 ms), *F*(1, 38) = 14.92, *p* < 0.001, \eta _{\rm{p}}^2 = 0.28. Effects involving form repetition were not significant, including the main effect of form repetition, *F*(1, 19) = 2.71, *p* = 0.113, \eta _{\rm{p}}^2 = 0.12, the interaction between location repetition and form repetition, *F*(1, 19) = 0.41, *p* = 0.535, \eta _{\rm{p}}^2 = 0.02, the interaction between form repetition and response repetition, *F*(1, 19) = 1.26, *p* = 0.275, \eta _{\rm{p}}^2 = 0.06, and the three-way interaction of all three variables, *F*(1, 19) = 0.51, *p* = 0.489, \eta _{\rm{p}}^2 = 0.03. A further Bayesian repeated-measures ANOVA (JASP Team, 2022) showed anecdotal evidence for the null model for the two-way interaction of form repetition and response repetition (BFincl = 0.624) and the three-way interaction of location repetition, form repetition, and response repetition (BFincl = 0.430). Other effects were not significant (*p* > 0.20).

An analysis of error rates revealed a significant interaction between location repetition and response repetition, *F*(1, 19) = 4.71, *p* = 0.041, \eta _{\rm{p}}^2 = 0.20. A follow-up simple main effect analysis indicated that location repetition produced a significant repetition benefit when the response was repeated (1.2%), *F*(1, 38) = 6.27, *p* = 0.016, \eta _{\rm{p}}^2 = 0.14, and a non-significant repetition cost when the response was not repeated (–0.2%), *F*(1, 38) = 0.23, *p* = 0.626, \eta _{\rm{p}}^2 = 0.01. Additionally, the main effect of the form was significant, *F*(1, 19) = 7.65, *p* = 0.012, \eta _{\rm{p}}^2 = 0.29, suggesting fewer errors when the form was repeated. Other effects involving form repetition were not significant, including the interaction between location repetition and form repetition, *F*(1, 19) = 2.76, *p* = 0.110, \eta _{\rm{p}}^2 = 0.13, the interaction between form repetition and response repetition, *F*(1, 19) = 3.02, *p* = 0.095, \eta _{\rm{p}}^2 = 0.14, and the three-way interaction of all three variables, *F*(1, 19) = 0.83, *p* = 0.376, \eta _{\rm{p}}^2 = 0.04. A further Bayesian repeated measures ANOVA showed anecdotal evidence for the null model for the two-way interaction of form and response repetition (BFincl = 0.517) and the three-way interaction of location, form and response repetition (BFincl = 0.487).

### Reaction time bin analysis

**Figure 3:**
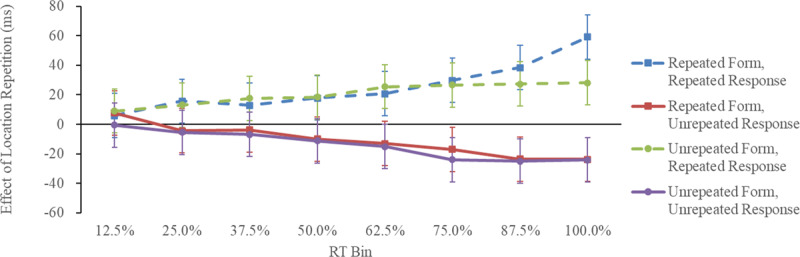
The Effect of Location Repetition as a Function of Form Repetition, Response Repetition, and RT Bin in Experiment 1 (Detection Task). *Note*: Effect of location repetition = unrepeated-location RT – repeated-location RT. Error bars represent 95% confidence intervals based on Jarmasz and Hollands (2009). The MSE was based on an ANOVA of the effect of the location repetition.

Table 2 lists each condition’s average median correct RTs and error rates. Figure 3 shows the effect of location repetition (i.e., unrepeated-location RT – repeated-location RT) as a function of form repetition, response repetition, and RT bin. The RT data were analyzed using location repetition (repeated/unrepeated) × form repetition (repeated/unrepeated) × response repetition (repeated/unrepeated) × RT bin (12.5%, 25%, 37.5%, 50%, 62.5%, 75%, 87.5%, and 100%) four-way repeated-measures ANOVA.

**Table 2:** Averaged Median Correct Response Times (RT, in ms) and Standard Deviations (in parentheses) as a Function of Location, Form, and Response Repetition and RT Bin in Experiment 1 (Detection Task).


RT BIN	LOCATION REPEATED				LOCATION UNREPEATED			
	
	FORM REPEATED		FORM UNREPEATED		FORM REPEATED		FORM UNREPEATED	
			
	RESPONSE REPEATED	RESPONSE UNREPEATED	RESPONSE REPEATED	RESPONSE UNREPEATED	RESPONSE REPEATED	RESPONSE UNREPEATED	RESPONSE REPEATED	RESPONSE UNREPEATED

12.5%	222(48)	219(47)	216(43)	207(59)	228(60)	227(50)	225(47)	207(54)

25%	261(31)	270(42)	263(25)	265(40)	277(36)	266(36)	276(31)	260(32)

37.5%	286(30)	297(34)	283(27)	294(37)	299(33)	293(36)	301(37)	287(27)

50%	306(35)	323(39)	305(31)	318(41)	323(40)	313(39)	323(45)	306(31)

62.5%	327(41)	349(52)	323(34)	342(52)	347(47)	336(49)	349(53)	327(38)

75%	347(46)	375(63)	349(48)	376(66)	377(51)	358(58)	376(62)	352(45)

87.5%	389(63)	423(81)	388(65)	421(76)	428(67)	399(70)	416(75)	396(68)

100%	479(100)	520(93)	469(87)	524(100)	538(106)	496(96)	497(99)	500(99)


The analysis revealed that the main effects of location repetition, *F*(1, 19) = 4.31, *p* = 0.049, \eta _{\rm{p}}^2 = 0.19, form repetition, *F*(1, 19) = 4.77, *p* = 0.040, \eta _{\rm{p}}^2 = 0.20, and RT bin, *F*(7, 133) = 181.00, *p* < 0.001, \eta _{\rm{p}}^2 = 0.91, were significant. Moreover, the two-way interactions of location repetition and response repetition, *F*(1, 19) = 40.05, *p* < 0.001, \eta _{\rm{p}}^2 = 0.68, and response repetition and RT bin, *F*(7, 133) = 2.23, *p* = 0.035, \eta _{\rm{p}}^2 = 0.11, also reached significance. More importantly, the three-way interactions of location repetition, response repetition, and RT bin, *F*(7, 133) = 7.98, *p* < 0.001, \eta _{\rm{p}}^2 = 0.30, and form repetition, response repetition, and RT bin, *F*(7, 133) = 4.28, *p* < 0.001, \eta _{\rm{p}}^2 = 0.18, were significant. A follow-up simple-simple main effect for the three-way interaction of location repetition, response repetition, and RT bin showed that the benefit of location repetition when the response was repeated was significant at 25%, *F*(1, 304) = 5.60, *p* = 0.018, \eta _{\rm{p}}^2 = 0.02, 37.5%, *F*(1, 304) = 6.35, *p* = 0.012, \eta _{\rm{p}}^2 = 0.02, 50%, *F*(1, 304) = 8.89, *p* = 0.004, \eta _{\rm{p}}^2 = 0.03, 62.5%, *F*(1, 304) = 14.52, *p* < 0.001, \eta _{\rm{p}}^2 = 0.05, 75%, *F*(1, 304) = 21.74, *p* < 0.001, \eta _{\rm{p}}^2 = 0.07, 87.5%, *F*(1, 304) = 29.44, *p* < 0.001, \eta _{\rm{p}}^2 = 0.09, and 100%, *F*(1, 304) = 51.91, *p* < 0.001, \eta _{\rm{p}}^2 = 0.15, RT bins. The cost of location repetition when the responses were not repeated was significant in the 62.5%, *F*(1, 304) = 5.39, *p* = 0.020, \eta _{\rm{p}}^2 = 0.02, 75%, *F*(1, 304) = 11.40, *p* = 0.001, \eta _{\rm{p}}^2 = 0.05, 87.5%, *F*(1, 304) = 15.92, *p* < 0.001, \eta _{\rm{p}}^2 = 0.05, and 100%, *F*(1, 304) = 15.69, *p* < 0.001, \eta _{\rm{p}}^2 = 0.05, RT bins. A follow-up simple-simple main effect for the three-way interaction of form repetition, response repetition, and RT bin suggested that there were significant form repetition costs when the response was repeated in the 100% RT bin, *F*(1, 304) = 23.29, *p* < 0.001, \eta _{\rm{p}}^2 = 0.07, and when the response was not repeated in the 12.5% RT bin, *F*(1, 304) = 8.94, *p* = 0.003, \eta _{\rm{p}}^2 = 0.03.

### Discussion

The present experiment’s findings are similar to those of Chao and Hsiao (2021), suggesting the integration of location and response. Therefore, there was a FOR effect (benefit of location repetition) when the response was repeated and an IOR effect (cost of location repetition) when the response was not repeated. These findings are consistent with predictions based on the concept of event files (Hommel 1998, 2004) and the BRAC framework (Frings et al. 2020).

Additionally, similar to Chao and Hsiao (2021), the findings of the present experiment show that the retrieval of previous episodic representations requires time. The RT bin analysis showed that the effects of both FOR and IOR were significant when RTs were relatively longer. Similarly, Schöpper and Frings (2022) found that the IOR was larger when RTs became longer. These findings suggest the importance of time-dependent retrieval processing of previous episodic representations to affect current performance (Frings et al. 2020).

An unexpected finding of this experiment was the significant three-way interaction between form repetition, response repetition, and RT bin. Based on the intentional weighting mechanism (Memelink & Hommel 2013) and the effects of task demand on the processing of event files (Hommel 2019; Hommel et al. 2014), we expected that the form of the target would be less likely to modulate performance in the present experiment. A possible mechanism for these findings is distractor-response binding (Frings, Rothermund & Wentura 2007). The form of the target can be viewed as a distractor dimension because it is task irrelevant. Due to the binding between the distractor form and the response, repetition of the form could facilitate performance when the response was also repeated and delayed performance when the response was not repeated. Nevertheless, it should be noted that the cost of form repetition was observed in two conditions: when the response was repeated in the 100% RT bin and when the response was not repeated in the 12.5% RT bin. This distractor-response binding hypothesis can account for when the response was not repeated but not when it was repeated.

Notably, such a form repetition cost was observed under limited conditions. Contrastingly, the FOR effect for location repetition in the repeated response condition was observed in seven successive RT bins (from 25% to 100%), and the IOR effect for location repetition in the unrepeated response condition was observed in four following conditions (from 62.5% to 100%). Hence, we speculate that the cost of form repetition observed in the present experiment might be a less robust phenomenon. This finding is examined further in Experiment 2, in which the target form is also task irrelevant.

## Experiment 2: Localization Task

In Experiment 2, target localization instead of target detection task was used. Hommel’s (1998) response-cuing procedure was also used to manipulate response repetition in this experiment. We expected that, similar to the findings in Experiment 1 and Chao and Hsiao (2021), the locations and responses would be integrated, and the effect of location repetition would be modulated by response repetition. Moreover, because the form of the target was still task-irrelevant in this experiment, we did not expect strong effects from form repetition.

### Participants

A total of 20 undergraduate students at Chung Yuan Christian University (3 men and 17 women, aged 20–22 years old, average age=20.6 years) participated in this experiment and received NT$320. The participants had a normal or corrected-to-normal vision. All participants provided written informed consent to participate in the study.

### Stimuli and procedure

The experimental design, stimuli, and procedure of Experiment 2 were identical to Experiment 1, with several exceptions. First, in Experiment 2, participants were instructed to indicate the target’s location by pressing the corresponding button. Second, at the beginning of each trial of each couplet, the placeholders were presented for 200 ms, followed by a 1,600-ms central cue. This central cue indicated either of the following: ‘When the target is presented at the left location, press the left button; when the target is presented at the right location, press the right button’. Or ‘when the target is presented at the left location, press the right button; when the target is presented at the right location, press the left button’. Third, the target was presented for 2,000 ms, or until a response was provided.

### Results

Responses to the second trial of each couplet were included in further analyses only when the responses to the first trial of the same couplet were correct. This criterion excluded 3.4% of the data (0.3%–13.5% per participant).

### Analysis of the overall data

Table 3 lists each condition’s average median correct RTs and error rates. Both RTs and error rates were analyzed using a location repetition (repeated/unrepeated) × form repetition (repeated/unrepeated) × response repetition (repeated/unrepeated) repeated-measures ANOVA.

**Table 3:** Averaged Median Correct Response Times (RT, in ms) and Error Rates (ER, %) and Standard Deviations (in parentheses) as a Function of Location, Form, and Response Repetition in Experiment 2 (Localization Task).


	LOCATION REPEATED				LOCATION UNREPEATED			
	
	FORM REPEATED		FORM UNREPEATED		FORM REPEATED		FORM UNREPEATED	
			
	RESPONSE REPEATED	RESPONSE UNREPEATED	RESPONSE REPEATED	RESPONSE UNREPEATED	RESPONSE REPEATED	RESPONSE UNREPEATED	RESPONSE REPEATED	RESPONSE UNREPEATED

RT	457(83)	508(108)	465(83)	519(100)	528(111)	478(100)	549(117)	488(106)

ER	1.2(2.1)	5.9(5.4)	1.1(1.5)	5.3(4.3)	5.0(4.7)	2.5(2.7)	6.2(6.3)	1.8(4.2)


**Figure 4:**
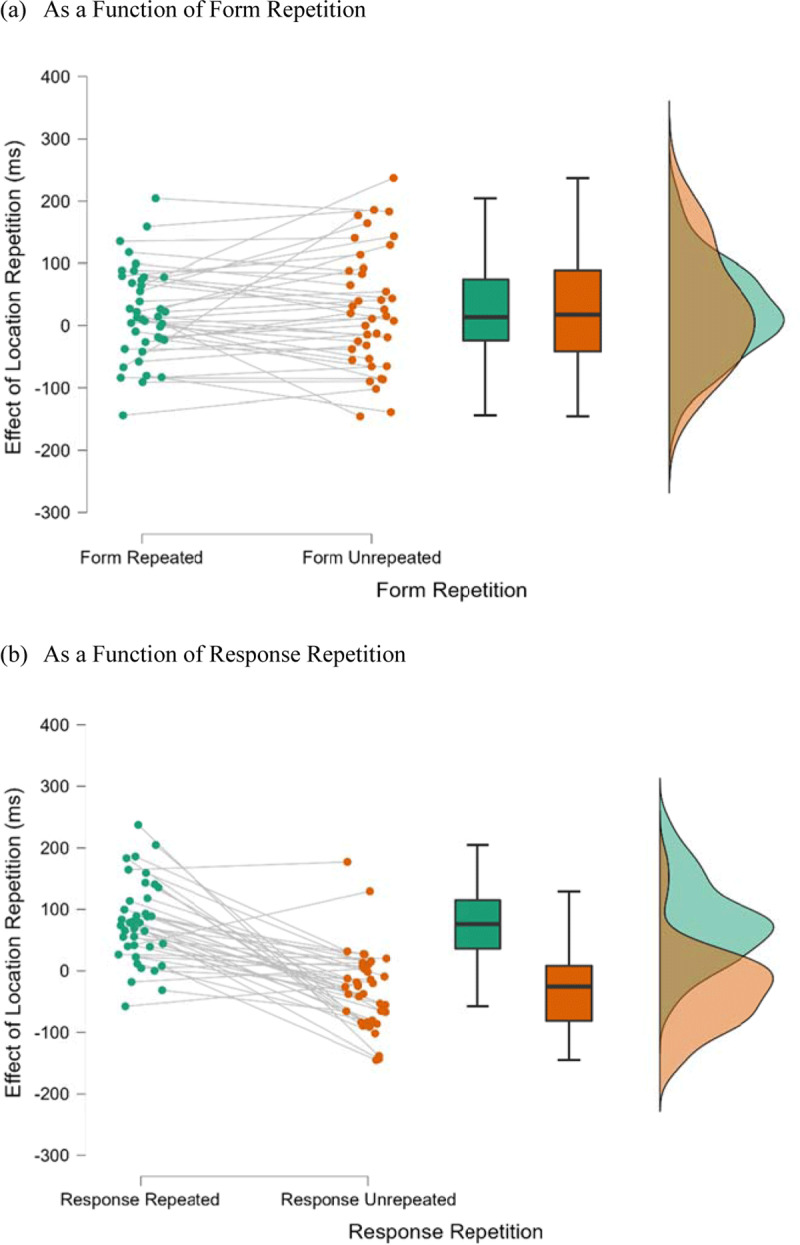
Raincloud Plots for the Effect of Location Repetition as a Function of Form Repetition and Response Repetition in Experiment 2 (Localization Task). *Note*: Effect of location repetition = unrepeated-location RT – repeated-location RT.

Analysis of the RT data showed significant main effects for location repetition, *F*(1, 19) = 8.75, *p* = 0.008, \eta _{\rm{p}}^2 = 0.32, and form repetition, *F*(1, 19) = 7.42, *p* = 0.013, \eta _{\rm{p}}^2 = 0.28. Moreover, the two-way interaction between location repetition and response repetition was significant, *F*(1, 19) = 31.82, *p* < 0.001, \eta _{\rm{p}}^2 = 0.63. Figure 4 shows the raincloud plots for the effect of location repetition as a function of response repetition and form repetition. A follow-up simple main effect analysis indicated that location repetition produced a significant repetition benefit when the response was repeated (77 ms), *F*(1, 38) = 38.85, *p* < 0.001, \eta _{\rm{p}}^2 = 0.51, and a significant repetition cost when the response was not repeated (–31 ms), *F*(1, 38) = 6.07, *p* = 0.017, \eta _{\rm{p}}^2 = 0.14. Interactions involving form repetition were not significant, including the interaction between location repetition and form repetition, *F*(1, 19) = 0.28, *p* = 0.600, \eta _{\rm{p}}^2 = 0.01, the interaction between form repetition and response repetition, *F*(1, 19) = 0.24, *p* = 0.622, \eta _{\rm{p}}^2 = 0.01, and the three-way interaction between location repetition, form repetition, and response repetition, *F*(1, 19) = 0.62, *p* = 0.448, \eta _{\rm{p}}^2 = 0.03. A further Bayesian repeated measures ANOVA showed anecdotal evidence for the null model for the two-way interaction of form and response repetition (BFincl = 0.391) and the three-way interaction of location repetition, form repetition, and response repetition (BFincl = 0.350). Other effects were not significant (*p* > 0.20).

The analysis of error rates revealed a significant interaction between location repetition and response repetition, *F*(1, 19) = 29.84, *p* < 0.001, \eta _{\rm{p}}^2 = 0.61. A follow-up simple main effect analysis indicated that location repetition produced a significant repetition benefit when the response was repeated (4.5%), *F*(1, 38) = 24.34, *p* < 0.001, \eta _{\rm{p}}^2 = 0.39, and a significant repetition cost when the response was not repeated (-3.4%), *F*(1, 38) = 14.23, *p* < 0.001, \eta _{\rm{p}}^2 = 0.27. Interactions involving form repetition were not significant, including the interaction between location repetition and form repetition, *F*(1, 19) = 0.41, *p* = 0.534, \eta _{\rm{p}}^2 = 0.02, the interaction between form repetition and response repetition, *F*(1, 19) = 2.24, *p* = 0.148, \eta _{\rm{p}}^2 = 0.11, and the three-way interaction of location repetition, form repetition, and response repetition, *F*(1, 19) = 0.65, *p* = 0.435, \eta _{\rm{p}}^2 = 0.03. A further Bayesian repeated measures ANOVA showed anecdotal evidence for the null model for the two-way interaction of form repetition and response repetition (BFincl = 0.537) and the three-way interaction of location repetition, form repetition, and response repetition (BFincl = 0.289). The other effects were not significant (*p* > 0.10).

### Reaction time bin analysis

**Figure 5:**
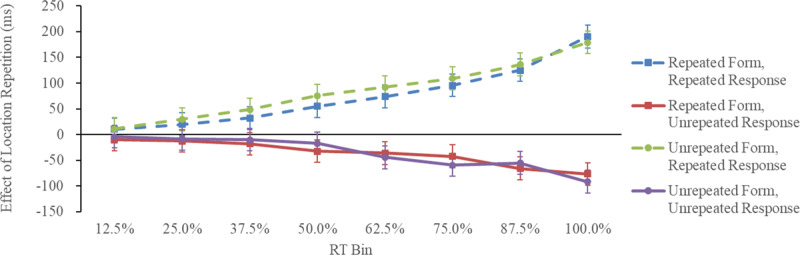
Effect of Location Repetition as a Function of Form Repetition, Response Repetition, and RT Bin in Experiment 2 (Localization Task). *Note*: Effect of location repetition = unrepeated-location RT – repeated-location RT. Error bars represent 95% confidence intervals based on Jarmasz and Hollands (2009). The MSE was based on an ANOVA of the effect of the location repetition.

Table 4 lists each condition’s average median correct RTs and error rates. Figure 5 shows the effect of location repetition as a function of form repetition, response repetition, and RT bin. The RT data were analyzed using location repetition (repeated/unrepeated) × form repetition (repeated/unrepeated) × response repetition (repeated/unrepeated) × RT bin (12.5%, 25%, 37.5%, 50%, 62.5%, 75%, 87.5%, and 100%) four-way repeated-measures ANOVA.

**Table 4:** Averaged Median Correct Response Times (RT, in ms) and Standard Deviations (in Parentheses) as a Function of Location, Form, and Response Repetition and RT Bin in Experiment 2 (Localization Task).


RT BIN	LOCATION REPEATED				LOCATION UNREPEATED			
	
	FORM REPEATED		FORM UNREPEATED		FORM REPEATED		FORM UNREPEATED	
			
	RESPONSE REPEATED	RESPONSE UNREPEATED	RESPONSE REPEATED	RESPONSE UNREPEATED	RESPONSE REPEATED	RESPONSE UNREPEATED	RESPONSE REPEATED	RESPONSE UNREPEATED

12.5%	350(56)	345(60)	348(63)	348(68)	360(73)	335(73)	359(76)	344(74)

25%	385(62)	396(73)	384(65)	398(71)	405(81)	384(83)	414(93)	390(84)

37.5%	412(71)	440(84)	412(70)	442(77)	444(93)	422(87)	461(99)	431(96)

50%	440(77)	488(99)	448(78)	488(89)	495(105)	456(91)	524(115)	471(104)

62.5%	481(91)	534(117)	484(86)	552(110)	555(115)	498(109)	576(123)	508(108)

75%	530(112)	602(126)	539(111)	621(117)	625(129)	560(126)	649(145)	561(116)

87.5%	614(139)	712(163)	618(147)	707(126)	739(148)	646(146)	754(175)	651(153)

100%	810(185)	892(223)	804(208)	940(189)	1001(237)	815(196)	983(249)	848(225)


The analysis revealed that the main effects of location repetition, *F*(1, 19) = 8.83, *p* = 0.008, \eta _{\rm{p}}^2 = 0.32, and RT bin, *F*(7, 133) = 176.68, *p* < 0.001, \eta _{\rm{p}}^2 = 0.90, were significant. Additionally, the two-way interactions of location repetition and response repetition, *F*(1, 19) = 51.91, *p* < 0.001, \eta _{\rm{p}}^2 = 0.73, location repetition and RT bin, *F*(7, 133) = 4.11, *p* < 0.001, \eta _{\rm{p}}^2 = 0.18, and response repetition and RT bin, *F*(7, 133) = 2.22, *p* = 0.036, \eta _{\rm{p}}^2 = 0.10, also reached significance.

Critically, the three-way interactions of location repetition, response repetition, and RT bin, *F*(7, 133) = 18.09, *p* < 0.001, \eta _{\rm{p}}^2 = 0.49, and form repetition, response repetition, and RT bin, *F*(7, 133) = 3.08, *p* = 0.005, \eta _{\rm{p}}^2 = 0.14, were significant. A follow-up simple-simple main effect analysis for the three-way interaction of location repetition, response repetition, and RT bin indicated that the benefit of location repetition when the response was repeated was significant in the 37.5%, *F*(1, 304) = 6.19, *p* = 0.013, \eta _{\rm{p}}^2 = 0.02, 50%, *F*(1, 304) = 16.44, *p* < 0.001, \eta _{\rm{p}}^2 = 0.05, 62.5%, *F*(1, 304) = 26.44, *p* < 0.001, \eta _{\rm{p}}^2 = 0.08, 75%, *F*(1, 304) = 40.43, *p* < 0.001, \eta _{\rm{p}}^2 = 0.12, 87.5%, *F*(1, 304) = 65.74, *p* < 0.001, \eta _{\rm{p}}^2 = 0.18, and 100%, *F*(1, 304) = 131.58, *p* < 0.001, \eta _{\rm{p}}^2 = 0.30, RT bins. Moreover, the cost of location repetition when the responses were not repeated was significant in the 62.5%, *F*(1, 304) = 6.27, *p* = 0.012, \eta _{\rm{p}}^2 = 0.02, 75%, *F*(1, 304) = 9.97, *p* = 0.002, \eta _{\rm{p}}^2 = 0.03, 87.5%, *F*(1, 304) = 14.18, *p* < 0.001, \eta _{\rm{p}}^2 = 0.04, and 100%, *F*(1, 304) = 27.44, *p* < 0.001, \eta _{\rm{p}}^2 = 0.08, RT bins. A follow-up simple-simple main effect analysis for the three-way interactions of form repetition, response repetition, and RT bin suggested that form repetition was significantly beneficial when the response was not repeated in the 100% RT bin, *F*(1, 304) = 14.53, *p* < 0.001, \eta _{\rm{p}}^2 = 0.05. The other effects were not significant (*p* > 0.10).

### Discussion

In Experiment 2, the localization task was used instead of the detection task. Findings similar to those of Experiment 1 and those of Chao and Hsiao (2021) were observed. First, there is clear evidence of the binding of locations and responses. Second, the effects of location repetition as a function of response repetition were observed in slower RT bins, suggesting that it takes time for memory retrieval.

Although there was an interaction between form repetition, response repetition, and RT bin, similar to Experiment 1, the benefit of form repetition was observed in one RT bin only. In contrast, the FOR effect for location repetition in the response-repeated condition was observed in six successive RT bins (from 37.5% to 100%), and the IOR effect for location repetition in the response-unrepeated condition was observed in four successive conditions (from 62.5% to 100%). Hence, the impact of form repetition was less robust in the present experiment.

Moreover, the significant cost of form repetition when the response was repeated in the 100% RT bin and when the response was not repeated in the 12.5% RT bin in Experiment 1 was not observed in Experiment 2. Hence, the cost of form repetition in Experiment 1 did not seem to be a robust phenomenon.

Finally, it should be noted that an additional analysis revealed that the compatibility between the target’s location and the response’s location interacted with other variables in this experiment (but not in Experiments 1 and 3). A location repetition (repeated/unrepeated) × form repetition (repeated/unrepeated) × response repetition (repeated/unrepeated) × compatibility (compatible/incompatible) repeated-measures ANOVA showed a significant interaction between form repetition, response repetition, and compatibility in Experiment 2, *F*(1, 19) = 7.31, *p* = 0.014, \eta _{\rm{p}}^2 = 0.28. A follow-up analysis of the simple-simple main effect showed that the compatibility effect, or the Simon-like effect, was significant when the form was repeated, but the response was not repeated, *F*(1, 76) = 8.15, *p* = 0.006, \eta _{\rm{p}}^2 = 0.10, and when the response was repeated but the form was not repeated, *F*(1, 76) = 7.26, *p* = 0.009, \eta _{\rm{p}}^2 = 0.09. The compatibility effect was not significant when both the form and response were repeated, *F*(1, 76) = 0.32, *p* = 0.578, \eta _{\rm{p}}^2 = 0.004, or when neither the form nor the response was repeated, *F*(1, 76) = 0.06, *p* = 0.682, \eta _{\rm{p}}^2 = 0.001. These findings imply that the Simon effect was easier to manifest when there was a partial match between the first and second trials. However, this finding requires further investigation because this interaction was only observed in Experiment 2.

## Experiment 3: Discrimination Task

In the third experiment, a target discrimination task was used. The participants were instructed to indicate the target form. As the form of the target was task-relevant, we expected that the form would be bound to the episodic representations and modulate performance in subsequent trials when part (or all) of the episode is repeated.

### Participants

A total of 20 undergraduate students at Chung Yuan Christian University (5 men and 15 women, aged 20–25 years old, average age = 21.2 years) participated in this experiment and received NT$320. The participants had a normal or corrected-to-normal vision. All participants provided written informed consent to participate in the study.

### Stimuli and procedure

The experimental design, stimuli, and procedure of Experiment 3 were identical to Experiment 1, with several exceptions. First, in Experiment 3, participants were instructed to indicate the form of the target by pressing the corresponding button. Second, at the beginning of each trial of each couplet, the central cue indicated either of the following: ‘When the target is an oval, press the left button; when the target is a rectangle, press the right button’. Or ‘when the target is an oval, press the right button; when the target is a rectangle, press the left button’. Third, the target was presented for 2,000 ms, or until a response was provided.

### Results

Responses to the second trial of each couplet were included in further analyses only when the responses to the first trial of the same couplet were correct. Based on this criterion, 8.9% of the data (1.3% to 20.6% per participant) were excluded.

### Analysis of the overall data

Table 5 lists each condition’s average median correct RTs and error rates. Both RTs and error rates were analyzed using location repetition (repeated/unrepeated) × form repetition (repeated/unrepeated) × response repetition (repeated/unrepeated) repeated-measures ANOVA.

**Table 5:** Averaged Median Correct Response Times (RT, in ms), Error Rates (ER, %), and Standard Deviations (in parentheses) as a Function of Location, Form, and Response Repetition in Experiment 3 (Discrimination Task).


	LOCATION REPEATED				LOCATION UNREPEATED			
	
	FORM REPEATED		FORM UNREPEATED		FORM REPEATED		FORM UNREPEATED	
			
	RESPONSE REPEATED	RESPONSE UNREPEATED	RESPONSE REPEATED	RESPONSE UNREPEATED	RESPONSE REPEATED	RESPONSE UNREPEATED	RESPONSE REPEATED	RESPONSE UNREPEATED

RT	569(98)	695(143)	744(122)	652(103)	628(117)	656(127)	761(139)	666(104)

ER	5.1(5.1)	10.2(7.7)	14.0(7.3)	6.1(4.8)	7.7(7.0)	6.5(6.1)	14.6(9.8)	6.2(6.0)


**Figure 6:**
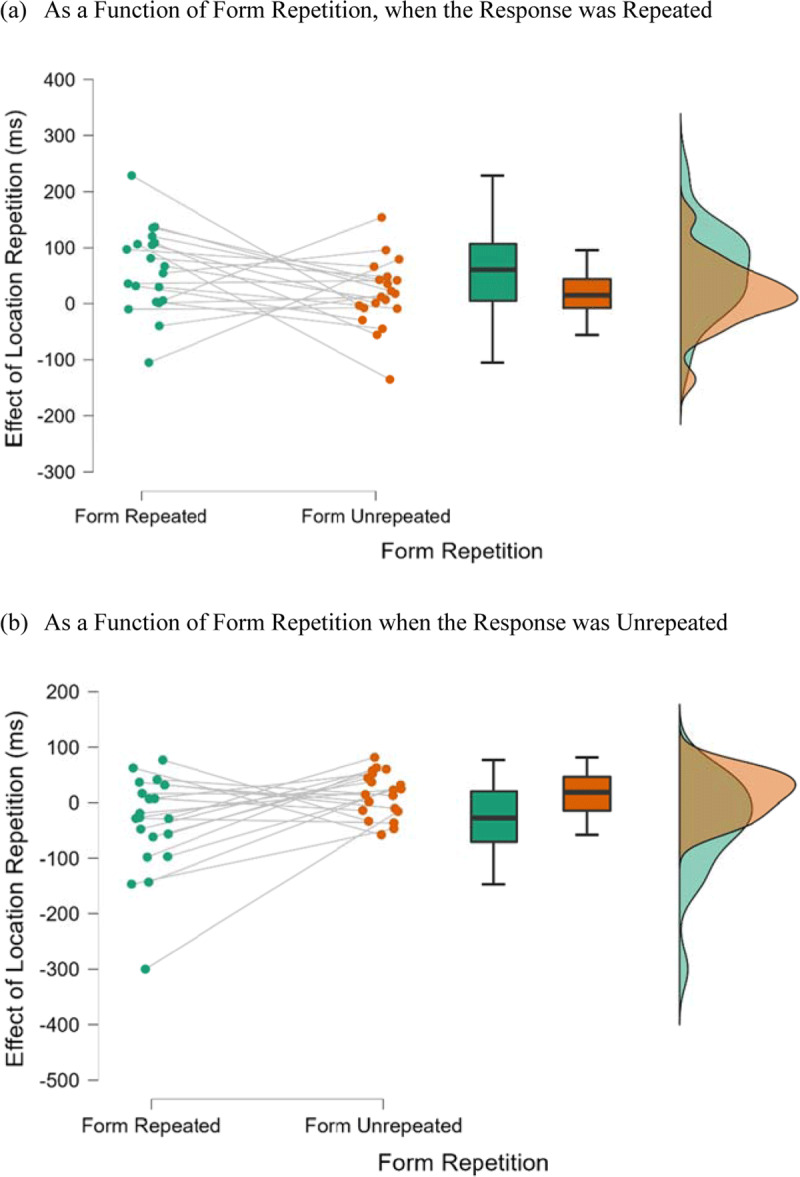
Raincloud Plots for the Effect of Location Repetition as a Function of Form Repetition When the Response was Repeated or Unrepeated in Experiment 3 (Discrimination Task). *Note*: Effect of location repetition = unrepeated-location RT – repeated-location RT.

Analysis of the RT data revealed a significant main effect of form repetition, *F*(1, 19) = 35.42, *p* < 0.001, \eta _{\rm{p}}^2 = 0.65. In this experiment, the main effect of location repetition was not significant, *F*(1, 19) = 2.95, *p* = 0.099, \eta _{\rm{p}}^2 = 0.13. The two-way interactions of location repetition and response repetition, *F*(1, 19) = 15.76, *p* = 0.001, \eta _{\rm{p}}^2 = 0.45, and form repetition and response repetition, *F*(1, 19) = 50.17, *p* < 0.001, \eta _{\rm{p}}^2 = 0.73, were both significant. More importantly, the three-way interaction of location repetition, form repetition, and response repetition was significant, *F*(1, 19) = 7.64, *p* = 0.012, \eta _{\rm{p}}^2 = 0.29. Figure 6 shows the raincloud plots for the effect of location repetition as a function of form repetition when the response was repeated or unrepeated. A follow-up simple-simple main effect analysis indicated that location repetition produced a repetition benefit when both the form and response were repeated (60 ms), *F*(1, 76) = 15.28, *p* < 0.001, \eta _{\rm{p}}^2 = 0.17, a repetition cost when the form was repeated and the response was not repeated (–39 ms), *F*(1, 76) = 6.45, *p* = 0.013, \eta _{\rm{p}}^2 = 0.08, and non-significant effects when the form was not repeated, regardless of whether the response was repeated (17 ms), *F*(1, 76) = 1.24, *p* = 0.267, \eta _{\rm{p}}^2 = 0.02, or not (14 ms), *F*(1, 76) = 0.89, *p* = 0.350, \eta _{\rm{p}}^2 = 0.01. Other effects were not significant (*p* > 0.20).

An analysis of error rates showed significant main effects of form repetition, *F*(1, 19) = 30.94, *p* < 0.001, \eta _{\rm{p}}^2 = 0.62, and response repetition, *F*(1, 19) = 30.92, *p* < 0.001, \eta _{\rm{p}}^2 = 0.62. Moreover, the two-way interactions of location repetition and response repetition, *F*(1, 19) = 11.08, *p* = 0.004, \eta _{\rm{p}}^2 = 0.37, and form repetition and response repetition, *F*(1, 19) = 24.21, *p* < 0.001, \eta _{\rm{p}}^2 = 0.56, were both significant. Finally, the three-way interaction of location repetition, form repetition, and response repetition was significant, *F*(1, 19) = 7.65, *p* = 0.012, \eta _{\rm{p}}^2 = 0.29. A follow-up simple-simple main effect analysis indicated that location repetition produced a repetition benefit when both the form and the response were repeated (2.6%), *F*(1, 76) = 4.22, *p* = 0.041, \eta _{\rm{p}}^2 = 0.05, a repetition cost when the form was repeated and the response was not (–3.7%_, *F*(1, 76) = 8.69, *p* = 0.005, \eta _{\rm{p}}^2 = 0.11, and non-significant effects when the form was not repeated, regardless of whether the response was repeated (0.63%), *F*(1, 76) = 0.25, *p* = 0.619, \eta _{\rm{p}}^2 < 0.01, or not (0.04%), *F*(1, 76) = 0.01, *p* = 0.434, \eta _{\rm{p}}^2 < 0.01. Other effects were not significant (*p* > 0.20).

### Reaction time bin analysis

**Figure 7:**
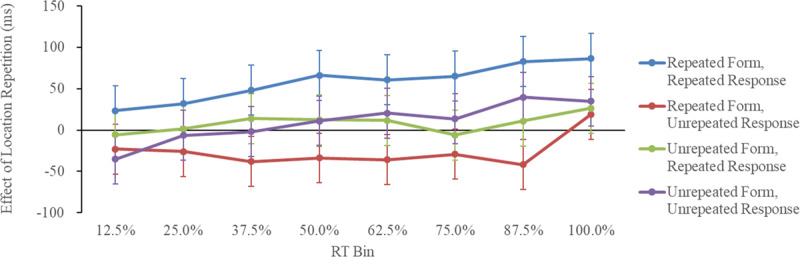
Effect of Location Repetition as a Function of Form Repetition, Response Repetition, and RT Bin in Experiment 3 (Discrimination Task). *Note*: Effect of location repetition = unrepeated-location RT – repeated-location RT. Error bars represent 95% confidence intervals based on Jarmasz and Hollands (2009). The MSE was based on an ANOVA of the effect of the location repetition.

Table 6 lists each condition’s average median correct RTs and error rates. Figure 7 shows the effect of location repetition as a function of form repetition, response repetition, and RT bin. The RT data were analyzed using location repetition (repeated/unrepeated) × form repetition (repeated/unrepeated) × response repetition (repeated/unrepeated) × RT bin (12.5%, 25%, 37.5%, 50%, 62.5%, 75%, 87.5%, and 100%) four-way repeated-measures ANOVA.

**Table 6:** Averaged Median Correct Response Times (RT, in ms) and Standard Deviations (in parentheses) as a Function of Location, Form, and Response Repetition and RT Bin in Experiment 3 (Discrimination Task).


RT BIN	LOCATION REPEATED				LOCATION UNREPEATED			
	
	FORM REPEATED		FORM UNREPEATED		FORM REPEATED		FORM UNREPEATED	
			
	RESPONSE REPEATED	RESPONSE UNREPEATED	RESPONSE REPEATED	RESPONSE UNREPEATED	RESPONSE REPEATED	RESPONSE UNREPEATED	RESPONSE REPEATED	RESPONSE UNREPEATED

12.5%	389(63)	428(82)	483(94)	463(86)	412(76)	405(79)	477(111)	427(77)

25%	442(73)	517(92)	566(104)	534(79)	474(85)	491(93)	567(123)	528(98)

37.5%	482(85)	591(105)	625(109)	580(92)	530(88)	552(108)	639(128)	578(99)

50%	531(89)	656(121)	708(114)	626(98)	597(111)	623(121)	720(136)	637(97)

62.5%	600(103)	730(150)	787(125)	675(109)	661(125)	694(132)	799(154)	695(108)

75%	591(134)	824(166)	891(166)	747(132)	756(137)	795(158)	885(175)	760(124)

87.5%	822(173)	952(186)	1022(204)	828(143)	905(174)	911(191)	1033(210)	868(167)

100%	1064(261)	1182(247)	1254(220)	1095(243)	1151(213)	1201(244)	1280(237)	1130(249)


The analysis revealed that the main effects of form repetition, *F* (1, 19) = 37.60, *p* < 0.001, \eta _{\rm{p}}^2 = 0.66, response repetition, *F*(1, 19) = 6.77, *p* = 0.017, \eta _{\rm{p}}^2 = 0.26, and RT bin, *F*(7, 133) = 287.23, *p* < 0.001, \eta _{\rm{p}}^2 = 0.94, were significant. The main effect of location repetition was not significant, *F*(1, 19) = 3.50, *p* = 0.074, \eta _{\rm{p}}^2 = 0.16. Additionally, the two-way interactions of location repetition and response repetition, *F*(1, 19) = 14.64, *p* = 0.001, \eta _{\rm{p}}^2 = 0.44, form repetition and response repetition, *F*(1, 19) = 54.34, *p* < 0.001, \eta _{\rm{p}}^2 = 0.74, location repetition and RT bin, *F*(7, 133) = 2.59, *p* = 0.016, \eta _{\rm{p}}^2 = 0.12, and response repetition and RT bin, *F*(7, 133) = 5.40, *p* < 0.001, \eta _{\rm{p}}^2 = 0.22, also reached significance.

More importantly, the three-way interactions of location repetition, form repetition, and response repetition, *F*(7, 133) = 9.46, *p* = 0.006, \eta _{\rm{p}}^2 = 0.33, and form repetition, response repetition, and RT bin, *F*(7, 133) = 17.01, *p* < 0.001, \eta _{\rm{p}}^2 = 0.47, were significant. A follow-up simple-simple main effect analysis for the three-way interaction of location repetition, form repetition, and response repetition indicated that location repetition produced a repetition benefit when both the form and the response were repeated, *F*(1, 76) = 21.35, *p* < 0.001, \eta _{\rm{p}}^2 = 0.22, a repetition cost when the form was repeated and the response was not, *F*(1, 76) = 4.34, *p* = 0.038, \eta _{\rm{p}}^2 = 0.05, and non-significant effects when the form was not repeated, regardless of whether the response was repeated, *F*(1, 76) = 0.41, *p* = 0.530, \eta _{\rm{p}}^2 < 0.01, or not, *F*(1, 76) = 0.56, *p* = 0.461, \eta _{\rm{p}}^2 < 0.01. A follow-up simple-simple main effect analysis for the three-way interaction of form repetition, response repetition, and RT bin suggests that the benefit of form repetition when the response was repeated was significant in the 12.5%, *F*(1, 304) = 16.60, *p* < 0.001, \eta _{\rm{p}}^2 = 0.05, 25%, *F*(1, 304) = 31.30, *p* < 0.001, \eta _{\rm{p}}^2 = 0.09, 37.5%, *F*(1, 304) = 41.82, *p* < 0.001, \eta _{\rm{p}}^2 = 0.12, 50%, *F*(1, 304) = 59.64, *p* < 0.001, \eta _{\rm{p}}^2 = 0.16, 62.5%, *F*(1, 304) = 69.49, *p* < 0.001, \eta _{\rm{p}}^2 = 0.19, 75%, *F*(1, 304) = 71.65, *p* < 0.001, \eta _{\rm{p}}^2 = 0.19, 87.5%, *F*(1, 304) = 71.71, *p* < 0.001, \eta _{\rm{p}}^2 = 0.19, and 100%, *F*(1, 304) = 67.38, *p* < 0.001, \eta _{\rm{p}}^2 = 0.18, RT bins. Moreover, the cost of form repetition when the responses were not repeated was significant in the 75%, *F*(1, 304) = 8.32, *p* = 0.005, \eta _{\rm{p}}^2 = 0.03, 87.5%, *F*(1, 304) = 18.58, *p* < 0.001, \eta _{\rm{p}}^2 = 0.06, and 100%, *F*(1, 304) = 16.38, *p* < 0.001, \eta _{\rm{p}}^2 = 0.05, RT bins.

Finally, the four-way interaction was not significant, *F*(7, 133) = 1.85, *p* = 0.083, \eta _{\rm{p}}^2 = 0.09. Other effects were not significant (*p* > 0.20).

## Discussion

In Experiment 3, unlike in Experiments 1 and 2, the form of the target was task relevant. In this task context, there was clear evidence that the form, location, and responses associated with the target were integrated. First, the three-way interaction of location repetition, form repetition, and response repetition in both RTs and error rates was significant. An additional four-way mixed ANOVA of experiment (Experiment 1/Experiment 2/Experiment 3) × location repetition (repeated/unrepeated) × form repetition (repeated/unrepeated) × response repetition (repeated/unrepeated) showed a significant four-way interaction, *F*(2, 57) = 6.95, *p* = 0.002, \eta _{\rm{p}}^2 = 0.20, in RTs, indicating that the three-way interaction of location repetition, form repetition, and response repetition was significant in Experiment 3, *F*(1, 57) = 16.88, *p* = 0.0003, \eta _{\rm{p}}^2 = 0.23, but not in Experiments 1, *F*(1, 57) = 0.06, *p* = 0.68, \eta _{\rm{p}}^2 = 0.001, and 2, *F*(1, 57) = 0.42, *p* = 0.53, \eta _{\rm{p}}^2 = 0.007. The three-way interaction of location repetition, form repetition, and response repetition indicated that location repetition produced a FOR effect (repetition benefit) when both the form and response were repeated, and an IOR effect (repetition cost) when the form was repeated and the response was not. Additionally, location repetition did not produce reliable results when the form was not repeated, regardless of whether the response was repeated. This finding supported the binding of a target event’s form, location, and response and the memory retrieval of the event representation when part of the features was repeated.

Moreover, RT bin analyses showed a three-way interaction between form repetition, response repetition, and RT bin. This interaction indicates that while the benefit of form repetition in the response-repeated condition was observed across all RT bins (from 12.5% to 100%), the cost of form repetition in the response-unrepeated condition was observed in longer RT bins (from 75% to 100%). This contrast suggests that the cost of form repetition might result from time-dependent memory retrieval of previous episodes. However, there was no clear evidence for the dependency of the benefit of form repetition on processing time. This contrast further suggests the need to investigate the underlying representations or mechanisms of benefits versus the cost of form repetition.

## General Discussion

The present study examined the binding of non-spatial features to location and response in the target-target paradigm of IOR (Maylor & Hockey 1985) across three experimental contexts: a target detection task in Experiment 1, a target localization task in Experiment 2, and a target discrimination task in Experiment 3. Consistent with the concept of event files (Hommel 1998, 2004), this study showed clear evidence for the binding of location and responses in Experiments 1 and 2 and the binding of form, location, and response in Experiment 3. For instance, there were FOR effects (benefit of location repetition) in the response-repeated condition and IOR effects (cost of location repetition) in the response-unrepeated condition in Experiments 1 and 2. Moreover, in Experiment 3, the FOR effect was contingent on repeating both forms and responses, and the IOR effect was contingent on form repetition. These findings were consistent with Huffman, Hilchey and Pratt’s (2018) review of the attentional-orienting literature and Schöpper et al.’s (2020) empirical findings. There was no evidence of non-spatial feature integration when a detection task or a localization task was used, and there was non-spatial feature integration when the non-spatial features were task-relevant, such as when a form discrimination task was sued.

The present study suggests that when processing a target event, the location, form, and response associated with the target can be integrated into an episodic representation or event file (Hommel 1998, 2004) of the target event. Thus, when some or all of the features are repeated later, they can serve as retrieval cues for the previous event files, and the retrieved event files can modulate the processing of the current target. When all features were repeated, there was a repetition benefit; when there was a partial match, there was a repetition cost (Milliken et al. 1998, 2000). These findings were also consistent with transfer-inappropriate and transfer-appropriate processing frameworks (Neill 2007; Neill & Mathis 1998).

In addition, according to the BRAC framework (Frings et al., 2020) and intentional weighting mechanism concept (Memelink & Hommel 2013), task demand might affect the binding or retrieval of event files. In the present study, there was clear evidence for the binding of the form and other features in Experiment 3, in which form discrimination was required. Contrastingly, there was less clear evidence for the binding of the form and other features in Experiments 1 and 2, in which form discrimination was not required. These findings are consistent with the BRAC framework and intentional weighting mechanism concept, suggesting the impact of task demand on the binding or retrieval of event files in the target-target paradigm of IOR.

It should be noted that because task demand remained the same across the entire experimental session, it is difficult to determine whether task demand modulates binding, retrieval, or both in the target-target paradigm of IOR. For instance, Hommel et al. (2014) demonstrated the effect of task demand on what was retrieved from event files. Moeller and Frings (2014) used a prime-probe paradigm and showed the importance of attention to probes, but not primes. Huffman, Hilchey and Pratt (2018) suggested that attention to forms and other features may be critical for feature binding. Various researchers have emphasized the impact of task demand on binding or retrieval. Therefore, we expect a future study in which the task demand for the first and second trials of the same couplet is manipulated independently (e.g., a detection task in the first trial and a discrimination task in the second) might be able to answer this critical question.

Moreover, memory retrieval is critical for the episodic representations, or the event files of the previous trials, to affect the processing of the current target (Frings et al. 2020). Thus, the present study showed that the FOR effects in the response-repeated condition and the IOR effects in the response-unrepeated condition were observed in longer RT bins in both Experiments 1 and 2. In Experiment 3, when a target discrimination task was used, the repetition cost of form repetition in the response-unrepeated condition was also observed in longer RT bins. These findings suggest that retrieval of previous episodic representations may require time to operate. These findings are consistent with and further generalize the findings of Chao and Hsiao (2021), who used a detection task. These findings are also consistent with Schöpper et al.’s (2020) hypothesis that memory retrieval is more likely to occur when responses are slow.

The findings of the present study suggest that, at least in the target-target paradigm of IOR, retrieval of previous event files significantly influences target processing, supporting the idea that the BRAC framework can explain findings in action control paradigms (Frings et al. 2020). These findings are also consistent with the constructive retrieval hypothesis (Milliken et al. 2000) and the detection cost hypothesis of IOR (Lupiáñez 2010; Lupiáñez et al. 2007; Lupiáñez, Martín-Arévalo & Chica 2013; Martín-Arévalo, Kingstone & Lupiáñez 2013), which proposed the roles of retrieved episodic representations. Furthermore, they suggested that the responses were integrated into the episodic representations of the target events and could be retrieved to modulate later performance. However, it should be noted that whether the present study’s findings can be applied to all IOR-related phenomena and paradigms requires further investigation. For instance, the present study showed the impact of task relevancy on the binding or retrieval of features (i.e., task-irrelevant features were less likely to affect performance). Nevertheless, task-irrelevant features can modulate performance in the cue-target paradigm of IOR (Hu, Samuel & Chan 2011; Klein et al. 2015). This suggests the possibility of different underlying mechanisms. Furthermore, it has been recently demonstrated that while binding in a detection task does not occur in the visual modality, it manifests in the auditory modality (Schöpper & Frings 2022). Hence, the effects of modality on the binding of location, form, and response in the target-target paradigm of IOR also require further investigation.

Finally, an alternative explanation deserves further consideration. In Experiment 2, when analyzing the effects of location repetition in each condition, the contrast between location repeated and location unrepeated co-varied with task repetition and task switching. Hence, this contrast may reflect the IOR, switch cost (Monsell 2003), or both. However, this potential confounding effect was not observed in Experiment 3. Although there was also a requirement for task switching, for the critical three-way interaction of location repetition, form repetition, and response repetition, task switching did not confound the contrast between location repeated and location unrepeated. For instance, when analyzing the effect of location repetition when both the form and response were repeated, there was no task switching in both the location repeated and location unrepeated conditions. When analyzing the effect of location repetition when the form was repeated and the response was not repeated, task switching was present in both the location repeated and location unrepeated conditions.

In summary, considering the effects of task switching, the interpretation of the findings of Experiment 2 was ambiguous. Regarding the interpretation of Experiment 2, the relationship between the findings of larger repetition effects when the response times were longer, and the effects of task switching require further investigation. On the one hand, the findings of Experiment 2 were not consistent with the findings of the distributional analyses of switch costs. For example, the numerical color-shape switch cost decreased in the final bin in Segal, Stasenko and Gollan’s (2019) study, and the local switch cost did not increase in the younger-adult group but decreased in the middle-aged and older adult groups in the final bin of Huff et al.’s (2015) study. Contrastingly, Experiment 2 results were similar to Experiment 1, in which task switching did not co-vary with location repetition and response repetition. On the other hand, according to the failure-to-engage hypothesis of residual switch costs (De Jong 2000), when there was a long time for task preparation, switch costs were especially expected when the response times were long. Hence, whether the findings of the distributional analyses of the present study can be explained by switch costs requires further investigation. Overall, task switching could partially explain the major findings of Experiment 2 but not Experiment 3. Therefore, the claim that the form was integrated with location and response when the form was task-relevant (Experiment 3) but not when the form was task-irrelevant (Experiment 1) was still supported by the present study’s findings. Further studies are required to investigate the role of task-switching in Experiments 2 and 3. For example, the binding of the response and other features in the target-target paradigm of IOR can be investigated in the context where a feature is task-relevant but response-irrelevant (Singh et al. 2018). For instance, this could be implemented with a task in which participants have to report the location or the form of the target in addition to the primary task requirement (a detection task with the manipulation of response repetition).

## Data Accessibility Statements

Data can be found at: https://osf.io/pcugr/.

## References

[1] Allen, M., Poggiali, D., Whitaker, K., Marshall, T. R., van Langen, J., & Kievit, R. A. (2021). Raincloud plots: a multi-platform tool for robust data visualization [version 2; peer review: 2 approved]. Wellcome Open Research, 4, 63. DOI: 10.12688/wellcomeopenres.15191.2 PMC6480976 31069261

[2] Chao, H. F., & Hsiao, F. S. (2021). Location-response binding and inhibition of return in a detection task. Attention, Perception, & Psychophysics, 83, 1992–2001. DOI: 10.3758/s13414-021-02283-4 33821452

[3] De Jong, R. (2000). An intention-activation account of residual switch costs. In S. Monsell & J. Driver (Eds.), Attention and performance XVIII: Control of cognitive processes (pp. 357–376). MIT Press.

[4] Dukewich, K. (2009). Reconceptualizing inhibition of return as habituation of the orienting response. Psychonomic Bulletin & Review, 16, 238–251. DOI: 10.3758/PBR.16.2.238 19293089

[5] Forster, K. I., & Forster, J. C. (2003). DMDX: A windows display program with millisecond accuracy. Behavior Research Methods, Instruments, & Computers, 35, 116–124. DOI: 10.3758/BF03195503 12723786

[6] Frings, C., Hommel, B., Koch, I., Rothermund, K., Dignath, D., Giesen, C., Kiesel, A., Kunde, W., Mayr, S., Moeller, B., Möller, M., Pfister, R., & Philipp, A. (2020). Binding and retrieval in action control (BRAC). Trends in Cognitive Sciences, 24, 375–387. DOI: 10.1016/j.tics.2020.02.004 32298623

[7] Frings, C., Rothermund, K., & Wentura, D. (2007). Distractor repetitions retrieve previous responses to targets. The Quarterly Journal of Experimental Psychology, 60, 1367–1377. DOI: 10.1080/17470210600955645 17853245

[8] Hommel, B. (1998). Event files: Evidence for automatic integration of stimulus–response episodes. Visual Cognition, 5, 183–216. DOI: 10.1080/713756773

[9] Hommel, B. (2004). Event files: Feature binding in and across perception and action. Trends in Cognitive Sciences, 8, 494–500. DOI: 10.1016/j.tics.2004.08.007 15491903

[10] Hommel, B. (2019). Theory of Event Coding (TEC) V2. 0: Representing and controlling perception and action. Attention, Perception, & Psychophysics, 81, 2139–2154. DOI: 10.3758/s13414-019-01779-4 PMC6848055 31168699

[11] Hommel, B., Memelink, J., Zmigrod, S., & Colzato, L. S. (2014). Attentional control of the creation and retrieval of stimulus–response bindings. Psychological Research, 78(4), 520–538. DOI: 10.1007/s00426-013-0503-y 23884516

[12] Hommel, B., Müsseler, J., Aschersleben, G., & Prinz, W. (2001). The Theory of Event Coding (TEC): A framework for perception and action planning. Behavioral and Brain Sciences, 24, 849–878. DOI: 10.1017/S0140525X01000103 12239891

[13] Hu, F. K., Samuel, A. G., & Chan, A. S. (2011). Eliminating inhibition of return by changing salient nonspatial attributes in a complex environment. Journal of Experimental Psychology: General, 140, 35–50. DOI: 10.1037/a0021091 21171801 PMC3051188

[14] Huff, M. J., Balota, D. A., Minear, M., Aschenbrenner, A. J., & Duchek, J. M. (2015). Dissociative global and local task-switching costs across younger adults, middle-aged adults, older adults, and very mild Alzheimer’s disease individuals. Psychology and Aging, 30(4), 727–739. DOI: 10.1037/pag0000057 26652720 PMC4681312

[15] Huffman, G., Hilchey, M. D., & Pratt, J. (2018). Feature integration in basic detection and localization tasks: Insights from the attentional orienting literature. Attention, Perception, & Psychophysics, 80, 1333–1341. DOI: 10.3758/s13414-018-1535-6 29717472

[16] Jarmasz, J., & Hollands, J. (2009). Confidence intervals in repeated-measures designs: The number of observations principle. Canadian Journal of Experimental Psychology, 63, 124–318. DOI: 10.1037/a0014164 19485604

[17] JASP Team. (2022). JASP (Version 0.16.3.0) [Computer software]. https://jasp-stats.org/

[18] Kahneman, D., Treisman, A., & Gibbs, B. J. (1992). The reviewing of object files: Object-specific integration of information. Cognitive Psychology, 24, 175–219. DOI: 10.1016/0010-0285(92)90007-O 1582172

[19] Klein, R. M. (2000). Inhibition of return. Trends in Cognitive Sciences, 4, 138–147. DOI: 10.1016/S1364-6613(00)01452-2 10740278

[20] Klein, R. M., Wang, Y., Dukewich, K. R., He, S., & Hu, K. (2015). On the costs and benefits of repeating a non-spatial feature in an exogenous spatial cuing paradigm. Attention, Perception, & Psychophysics, 77, 2293–2304. DOI: 10.3758/s13414-015-0941-2 26153659

[21] Lakens, D., & Caldwell, A. R. (2019). Simulation-Based Power-Analysis for Factorial ANOVA Designs. DOI: 10.31234/osf.io/baxsf

[22] Lupiáñez, J. (2010). Inhibition of return. In A. C. Nobre & J. T. Coull (Eds.), Attention and time (pp. 17–34). Oxford University Press. DOI: 10.1093/acprof:oso/9780199563456.001.0001

[23] Lupiáñez, J., Martín-Arévalo, E., & Chica, A. B. (2013). Is inhibition of return due to attentional disengagement or to a detection cost? The detection cost theory of IOR. Psicológica, 34, 221–252.

[24] Lupianez, J., Ruz, M., Funes, M. J., & Milliken, B. (2007). The manifestation of attentional capture: Facilitation or IOR depending on task demands. Psychological Research, 71(1), 77–91. DOI: 10.1007/s00426-005-0037-z 16333663

[25] Martín-Arévalo, E., Kingstone, A., & Lupiáñez, J. (2013). Is “Inhibition of Return” due to the inhibition of the return of attention? The Quarterly Journal of Experimental Psychology, 66, 347–359. DOI: 10.1080/17470218.2012.711844 22928599

[26] Maylor, E. A., & Hockey, R. (1985). Inhibitory component of externally controlled covert orienting in visual space. Journal of Experimental Psychology: Human Perception and Performance, 11, 777–787. DOI: 10.1037/0096-1523.11.6.777 2934508

[27] Memelink, J., & Hommel, B. (2013). Intentional weighting: A basic principle in cognitive control. Psychological Research, 77, 249–259. DOI: 10.1007/s00426-012-0435-y 22526717 PMC3627030

[28] Milliken, B., Joordens, S., Merikle, P. M., & Seiffert, A. E. (1998). Selective attention: A reevaluation of the implications of negative priming. Psychological Review, 105, 203–229. DOI: 10.1037/0033-295X.105.2.203 9577237

[29] Milliken, B., Tipper, S. P., Houghton, G., & Lupiáñez, J. (2000). Attending, ignoring, and repetition: On the relation between negative priming and inhibition of return. Perception & Psychophysics, 62, 1280–1296. DOI: 10.3758/BF03212130 11019624

[30] Mocke, V., Weller, L., Frings, C., Rothermund, K., & Kunde, W. (2020). Task relevance determines binding of effect features in action planning. Attention, Perception, & Psychophysics, 82, 3811–3831. DOI: 10.3758/s13414-020-02123-x PMC7593314 32914340

[31] Moeller, B., & Frings, C. (2014). Attention meets binding: Only attended distractors are used for the retrieval of event files. Attention, Perception, & Psychophysics, 76, 959–978. DOI: 10.3758/s13414-014-0648-9 24627211

[32] Monsell, S. (2003). Task switching. Trends in Cognitive Sciences, 7, 134–140. DOI: 10.1016/S1364-6613(03)00028-7 12639695

[33] Neill, W. T. (2007). Mechanisms of transfer-inappropriate processing. In D. S. Gorfein & C. M. MacLeod (Eds.), Inhibition in cognition (pp. 63–78). Washington, DC: American Psychological Association. DOI: 10.1037/11587-004

[34] Neill, W. T., & Mathis, K. M. (1998). Transfer-inappropriate processing: Negative priming and related phenomena. In D. L. Medin (Ed.), The psychology of learning and motivation: Advances in research and theory, 38, 1–44. Academic Press. DOI: 10.1016/S0079-7421(08)60182-6

[35] Posner, M. I., & Cohen, Y. (1984). Components of visual orienting. In H. Bouma and D. G. Bouwhuis (Eds.), Attention and performance X: Control of language processes (pp. 531–556). Erlbaum.

[36] Posner, M. I., Rafal, R. D., Choate, L. S., & Vaughan, J. (1985). Inhibition of return: Neural basis and function. Cognitive Neuropsychology, 2, 211–228. DOI: 10.1080/02643298508252866

[37] Ratcliff, R. (1979). Group reaction time distribution and an analysis of distribution statistics. Psychological Bulletin, 86, 446–461. DOI: 10.1037/0033-2909.86.3.446 451109

[38] Schöpper, L. M., & Frings, C. (2022). Same, but different: Binding effects in auditory, but not visual detection performance. Attention, Perception, & Psychophysics. DOI: 10.3758/s13414-021-02436-5 PMC9935720 35107812

[39] Schöpper, L. M., Hilchey, M. D., Lappe, M., & Frings, C. (2020). Detection versus discrimination: The limits of binding accounts in action control. Attention, Perception, & Psychophysics, 82, 2085–2097. DOI: 10.3758/s13414-019-01911-4 31823230

[40] Segal, D., Stasenko, A., & Gollan, T. H. (2019). More evidence that a switch is not (always) a switch: Binning bilinguals reveals dissociations between task and language switching. Journal of Experimental Psychology: General, 148, 501–519. DOI: 10.1037/xge0000515 30394767 PMC6389445

[41] Singh, T., Moeller, B., Koch, I., & Frings, C. (2018). May I have your attention please: Binding of attended but response-irrelevant features. Attention, Perception, & Psychophysics, 80, 1143–1156. DOI: 10.3758/s13414-018-1498-7 29520713

